# Whole Health coaching to rural Veterans through telehealth: Advantages, gaps, and opportunities

**DOI:** 10.3389/fpubh.2023.1057586

**Published:** 2023-03-27

**Authors:** Kelsea LeBeau, Deepthi S. Varma, Consuelo M. Kreider, Gail Castañeda, Cheri Knecht, Diane Cowper Ripley, Huanguang Jia, J. Hale-Gallardo

**Affiliations:** ^1^Veterans Rural Health Resource Center-Gainesville (VRHRC-GNV), Office of Rural Health, Veterans Health Administration, Gainesville, FL, United States; ^2^Department of Epidemiology, University of Florida, Gainesville, FL, United States; ^3^Department of Occupational Therapy, University of Florida, Gainesville, FL, United States; ^4^Director Emeritus, GeoSpatial Outcomes Division, Office of Rural Health, Veterans Health Administration, Gainesville, FL, United States; ^5^Department of Biostatistics, University of Florida, Gainesville, FL, United States; ^6^Veterans Rural Health Resource Center-Salt Lake City (VRHRC-SLC), Office of Rural Health, Veterans Health Administration, Salt Lake City, UT, United States

**Keywords:** telehealth, Veterans, health coaching, mixed methods, access to care, qualitative evaluation, health care delivery

## Abstract

**Background:**

The Veterans Health Administration (VHA) is one of the largest providers of telehealth in the United States and continues to lead the way in transforming healthcare services. VHA has been implementing its Whole Health (WH) initiative since 2018, a proactive practice empowering patients to take charge of their health and well-being. A key facilitator of the WH initiative is the WH coach who partners with Veterans to achieve their health-related goals. A gap exists in the literature regarding the understanding of WH coaches’ use of telehealth to engage rural-residing Veterans. COVID-19 unexpectedly interrupted in-person VHA delivery of care, including WH coaching which primarily relied on in-person delivery and focused less on telehealth. During the pandemic, WH coaches had to adapt and integrate different modalities to engage their Veteran patients. We examined WH coaches’ approaches to extending coaching to rural Veterans via technology, emphasizing the advantages of telehealth, existing gaps in telehealth delivery, and opportunities for telehealth as a coaching modality.

**Methods:**

This project was implemented as part of a larger mixed methods evaluation regarding WH coaching for rural Veterans; this manuscript presents the findings from the qualitative data from the larger study. The qualitative dataset is comprised of data collected using three different qualitative methods: four focus groups (*n* = 11; 3–4 participants per group), in-depth individual interviews (*n* = 9), and open-ended responses from a national web-based survey (*n* = 140). Focus group, in-depth interview, and open-ended survey data were collected sequentially and separately analyzed following each wave of data collection. Findings from the three analyses were then collaboratively merged, compared, reorganized, and refined by the evaluation team to create final themes.

**Results:**

Three final themes that emerged from the merged data were: (1) Advantages of Telehealth; (2) Telehealth Gaps for Rural Veterans, and (3) Strategies for Bridging Telehealth Gaps. Themes explicate telehealth advantages, gaps, and opportunities for rural Veteran WH coaching.

**Conclusion:**

Findings highlight that video telehealth alone is not sufficient for meeting the needs of rural Veterans. Digital technologies hold promise for equalizing health access gaps; however, both human factors and broadband infrastructure constraints continue to require WH coaches to use a mix of modalities in working with rural Veterans. To overcome challenges and bridge gaps, WH coaches should be ready to adopt a blended approach that integrates virtual, in-person, and lower-tech options.

## 1. Introduction

The Veterans Health Administration (VHA) of the Department of Veterans Affairs (VA) is one of the largest providers of telehealth in the United States (1). Their practices, initiatives, and health care innovations cut across other healthcare systems (2) and impact more than nine million enrolled Veterans (3). The VHA has increased equitable access to care for Veterans using telehealth, supplementing many face-to-face visits (4, 5). During COVID-19, VHA’s virtual delivery of healthcare grew exponentially, with some providers and patients having limited telehealth experience now participating in it. We are now witnessing the result of this expansion: a “new normal” wherein telehealth and remote modalities are being utilized to ensure continuous and accessible care for VHA enrolled Veterans (6, 7).

This virtual shift is especially important for rural Veterans as this population does not always have the same access to health care as their urban counterparts, despite more rural Veterans being enrolled in VHA healthcare (8, 9). Use of technology (e.g., web-based programs and video-delivered therapies) can help rural Veterans overcome barriers to accessing in-person care, such as long travel distances, closures of rural clinics, provider shortages, and long wait times (5, 10–13). However, virtual care delivery also comes with its own challenges, especially given the lack of access to broadband internet in rural communities (1, 12, 14, 15).

Since 2018, the VHA has been transforming its approach to care through the Whole Health (WH) initiative (16); a system-wide initiative that is being shepherded by the Office of Patient Centered Care and Cultural Transformation (OPCC&CT). WH is a proactive, integrative health approach empowering patients to take charge of their health through patient-centric decision-making rather than focusing primarily on treating disease (16–18). A key component of the WH initiative is the WH coach who assists Veterans in realizing their personalized health and wellbeing goals based on the Veteran’s desires, aspirations, and purpose (16, 19). In doing so, they bridge the gap between medical recommendations and Veterans’ abilities and inclinations to implement these recommendations. WH coaches collaborate closely with clinical teams, help Veterans with desired behavior changes, and facilitate engagement in relevant WH courses (e.g., “Intro to Whole Health” and “Taking Charge of My Life and Health”) (20). Many WH coaches work with rural Veterans, a hard-to-reach population facing unique barriers and constraints regarding healthcare access (8, 21–23).

Studies have demonstrated the integral role health coaching plays in patients achieving behavioral changes (24–27), and data show promising results for mental health, diabetes, heart disease, and general improvement of health and health-related quality of life (16, 20, 28–30). Prior to COVID-19, most WH coaching was conducted face-to-face with little integration of video telehealth coaching. However, virtual delivery of health coaching programs has been shown to be an effective approach and widespread supplement to in-person coaching, especially to increase access to care and resolve access barriers for people in rural areas (31).

While there is a growing body of literature focusing on health coaching and its virtual delivery (31, 32), a gap exists regarding our understanding of WH coaches’ use of telehealth to engage rural Veterans, and how different modalities are being used by WH coaches to meet the needs of these Veterans. The COVID-19 pandemic unexpectedly interrupted in-person delivery of care across VHA, including WH coaching which primarily relied on in-person delivery and focused much less on telehealth. During this transition, WH coaches had to adapt and integrate different modalities to engage with their Veteran patients. Therefore, the purpose of this paper was to examine WH coaches’ experiences with extending coaching to rural Veterans *via* technology, with an emphasis on explicating advantages of telehealth, existing gaps in telehealth delivery, and opportunities for telehealth as a coaching modality.

## 2. Materials and methods

### 2.1. Evaluation design

This project was implemented as part of a larger mixed methods evaluation regarding WH coaching for rural Veterans and was conceptualized by a multidisciplinary team. It was designed as a quality improvement project. As such, its ethical review and informed consent were waived according to VA’s relevant guideline (ORD Program Guide 1200.21).

We used multiple qualitative data collection techniques implemented over three waves of data collection, including focus group (FG) discussions, in-depth interviews, and open-ended survey responses from a national web-based survey, to understand WH coaches’ experiences with extending coaching to rural Veterans remotely. Each successive stage built on the previous, with the FGs providing a collective overview of how coaches perceived their ability to connect to rural Veterans at-a-distance, followed by in-depth interviews which permitted more in-depth exploration of how coaches were reaching rural Veterans, and finally the web-based survey, which allowed us to capture broader trends on barriers and facilitators to reaching rural Veterans with WH coaching.

### 2.2. Data collection and analysis

#### 2.2.1. Focus groups

During a June 2020 WH Community of Practice (COP) virtual call, 33 WH coaches were formally invited to participate in FGs regarding their outreach to rural Veterans. Two additional WH coaches were referred for FG participation by WH leadership, and a total of 11 coaches participated in three FGs (3–5 WH coaches in each group).

FG guides were prepared in dialogue with WH leadership and explored differences that coaches perceived in serving rural vs. non-rural Veterans and barriers and facilitators to coaching rural Veterans (Table 1). FGs were conducted virtually using Zoom and audio recorded with consent.

**Table 1:** Questions for focus groups, in-depth interviews, and open-ended surveys.

Focus group guide	
Question number	Questions and probes
1	In your WH coaching experience, what are some of the differences in providing WH coaching to rural Veterans versus urban Veterans? [Probe]: How is your coaching interaction different with rural vs. urban Veterans? [Probe]: What changes have you made to your coaching for working with rural Veterans? [Probe]: What factors may make programs more appealing for rural Veterans?
2	What are some of the barriers—or things that make it more difficult—to reach rural Veterans for WH?
3	What are some of the facilitators—or things that make it easier—to reach rural Veterans for WH?
4	Overall, do you have any special recommendations for how to reach and serve rural Veterans better?
In-depth interview guide	
Question number	Questions and probes
1	As a WH coach, please describe any differences you have experienced while providing WH coaching to rural versus urban Veterans? [Probe]: How is your coaching interaction different with rural vs. urban Veterans? [Probe]: What changes have you made to your coaching for working with rural Veterans? [Probe]: What factors may make programs more appealing for rural Veterans?
2	Please describe your process for recruiting rural Veterans into WH.
3	Please describe and provide examples of specific ways in which you modify your approach for extending WH to rural Veterans.
4	What are some of the barriers—or things that make it more difficult—to reach rural Veterans for WH?
5	What are some of the facilitators—or things that make it easier—to reach rural Veterans for WH?
6	Do you have any special recommendations for how to reach and serve rural Veterans better? [Probe]: WH Orientation? Technology—VVC or other? Knitting natural social connections?
7	What would you have liked us to ask you that we did not ask you?
Open-ended surveys	
Question number	Questions and probes
1	Please list any other of your duties as a WH Coach.
2	Please explain the differences you have perceived in tailoring your WH outreach approach for rural Veterans versus other Veterans in general.
3	Please list any other technological barriers you have encountered over the course of your WH outreach to rural Veterans.
4	Please list any other geographical barriers you have encountered over the course of your WH outreach to rural Veterans.
5	Please list any other cultural barriers you have encountered over the course of your WH outreach to rural Veterans.
6	Please list any other access barriers you have encountered over the course of your WH outreach to rural Veterans.
7	Please list any other types of barriers you have encountered over the course of your WH outreach to rural Veterans.
8	With regard to the barriers encountered over the course of your WH outreach to rural Veterans, what additional resources would help you overcome these barriers?
9	Please list what other factors has made it easier for you to reach rural Veterans.
10	Please list any other outreach strategies you have put into practice over the course of your WH outreach to rural Veterans.
11	Please list any other outreach strategies you have found to be MOST EFFECTIVE over the course of your WH outreach to rural Veterans.
12	Please list any other outreach strategies you have found to be LEAST EFFECTIVE over the course of your WH outreach to rural Veterans.
13	What is your favorite tool or strategy for reaching rural Veterans with WH? Why is this your favorite tool or strategy for reaching rural Veterans with WH? Please describe.

Audio recordings were sent to the Centralized Transcription Service Program (CTSP) for transcription. Transcribed data were analyzed with assistance of qualitative data analysis software. We used a “hybrid” approach in our coding process, beginning with a list of *a priori* codes developed based on the FG guide. New or emerging codes were then added as we explored the data inductively. After the first cycle of coding, we reviewed all codes to identify and compile groups of codes that reflected patterns or similar meanings; these groups of codes were categorized under themes that corresponded to the evaluation objectives. These initial themes exposed the need for a more in-depth exploration into how coaches were reaching rural Veterans with WH.

#### 2.2.2. In-depth interviews

In-depth interviews were conducted to allow for open-ended probing and exploration in a more private one-on-one environment to follow up on our preliminary understanding of how WH coaches were reaching rural Veterans and to confirm initial FG themes and domains. We used a convenience sample to recruit FG participants for the individual interviews through e-mail invitations sent to all FG participants. Among the 11 individuals invited, 9 agreed to participate.

We used the semi-structured interview guide that was developed from preliminary understandings from FGs (Table 1). This guide included questions that continued to inductively explore differences in coaching rural and urban Veterans, processes for recruiting rural Veterans into WH, and barriers and facilitators for reaching and serving rural Veterans. Interviews were *via* Zoom calls, and participants provided verbal consent to participate and be audio recorded.

Data from in-depth interviews were also transcribed using CTSP and analyzed with assistance of qualitative data analysis software. Analysis of in-depth interviews focused on gaining greater understanding of initial themes that emerged from FGs. The focus of the analysis was to explore and understand differences in WH coaching experiences between rural and urban Veterans, and specific strategies used to engage successfully with rural Veterans. Survey questions were then developed from these themes.

#### 2.2.3. Open-ended survey responses

A national survey was designed to describe WH coaching within the VHA health care system. The national survey was comprised of a total of 45 closed- and open-ended questions based on items generated from previous project phases (FGs and interviews). Open-ended survey questions asked about WH coaches’ duties and experiences reaching out to and coaching rural vs. non-rural Veterans (Table 1). The survey was used to explicate broader trends on barriers and facilitators to reaching rural Veterans with WH coaching, to check the breadth of our understanding of the topics generated from the FG discussions and interviews, and to understand the generalizability of these issues. Our survey was administered to a representative sample of VHA WH coaches nationwide *via* the WH coaching COP distribution list.

We obtained survey approval from VHA’s Organizational Assessment Committee in December 2020. Approval for survey dissemination was obtained in January 2021 from the American Federation of Government Employees union. Once finalized, the survey was disseminated *via* the COP distribution list. The survey allowed for individuals that did not meet eligibility criteria to exit before completion. Survey data were securely housed in the VA REDCap system (33, 34). Participation was voluntary and expected completion time was 20–25 min.

For the analysis reported here, we only included responses to the open-ended portion of the survey (comprising 13 open-ended questions from the larger 45-question survey). Open-ended survey data were initially analyzed using thematic analysis by three independent coders. Codes were developed both deductively from survey questions and inductively from survey data. The focus of this initial analysis included examination of differences in WH coaching between rural and urban Veterans, identification of specific strategies used to engage rural Veterans in WH, and preliminary conceptualization of alignment with our understandings from FGs and interviews.

### 2.3. Theme development from the three qualitative data sources

Following completion of the three sequential analyses, a merged analysis was conducted by reorganizing and collapsing conceptually similar themes from all three qualitative data sources. For this merged analysis and reorganization, the multidisciplinary evaluation team compared and reviewed initial themes from the three data sources, with a focus on telehealth for WH coaching with rural Veterans. Multiple rounds of discussion, comparison, and reorganization occurred before finalizing themes and subthemes. The final themes, which answered our *a priori* questions, were identified collaboratively by the evaluation team. Equal importance was given to themes common in all three datasets, and those that were outliers in each of the datasets were retained when they provided a nuanced understanding of the phenomenon. Codes and corresponding quotations were retrieved that reflected the identified themes from the FGs, in-depth interviews, and open-ended survey responses.

### 2.4. Analytic rigor

The approaches used in this evaluation were complementary to one another, which allowed for a comprehensive understanding of the phenomenon. Analytic rigor was further enhanced by our use of two triangulation strategies. Our first strategy was data source triangulation, which involves the use of different sources of data in the analysis, and the comparison and cross-checking of the consistency of information derived from the data sources, typically “by different means within qualitative methods” (35, 36). Our second strategy was investigator triangulation, which involves the “participation of two or more researchers in the same study” to conduct data analysis (35, 36). Additionally, analytic rigor was contributed to by the evaluation team’s sustained engagement with WH coaches and multiple instances of member checking across the larger evaluation.

## 3. Results

We had a total of 11 focus group participants (from 3 focus groups), 9 interview participants, and 140 survey participants. Participants shared experiences and perceptions of their WH practice during and after the COVID-19 pandemic, with telehealth emerging as a critical practice when working with rural Veterans. The main themes that emerged as salient to WH coaches included: (1) Advantages of Telehealth, (2) Telehealth Gaps for Rural Veterans, and (3) Strategies for Bridging Telehealth Gaps. Below is an in-depth explanation of the three themes and accompanying subthemes (Table 2).

**Table 2:** Final themes and subthemes from merged analysis of focus group, interview, and survey data.

Themes	Subthemes
Advantages of Telehealth	Increased Veteran participation
	Overcoming geographic barriers
	Convenience and flexibility
Telehealth Gaps for Rural Veterans	Rural internet access and connectivity issues
	Differing levels of digital literacy
	Receptivity to video telehealth coaching
Strategies for Bridging Telehealth Gaps	Increasing Veteran’s technological literacy
	Providing VHA-issued devices and virtual platforms
	Increasing access to technical support for Veterans and coaches
	Leveraging low-tech modalities
	Persisting relevance of in-person visits for rural Veterans

### 3.1. Advantages of telehealth

WH coaches reported several advantages of using telehealth as a modality to engage Veterans and work toward their health goals. Advantages included increased Veteran participation, overcoming geographic barriers, and convenience and flexibility.

#### 3.1.1. Increased veteran participation

Coaches noted that WH participation was increased by using telehealth to engage rural Veterans. Instead of relying primarily on in-person delivery, coaches perceived that telehealth allowed for more opportunities for WH involvement, especially following COVID-19 as efforts transitioned to being delivered *via* telehealth. One participant explained:

*[Rural Veterans] have an option [to] do the virtual group, or you have the option to come in…so I think in that context the rural individuals will have a better opportunity to participate. (Interview ID #15)*

Another participant described their success offering classes *via* VHA’s secure telehealth platform, Veterans Video Connect (VVC), as it permitted an expansion of capacity to more than double what was available in-person. For example, they mentioned increased participation, increased number of class offerings, and increased accessibility:

*[We offer Tai Chi] maybe like four or five times a week through VVC… [VVC] can hold up to say 30 Veterans versus we could only fit 12 people in a room…/… Currently, with the mindfulness classes on VVC, I might see six or seven Veterans on a Monday, another five or six on a Tuesday, and then I have [a class] on Thursdays…I think those numbers have gone up just ‘cause there is a lot more accessibility to them. (Interview ID #13)*

One coach explained telehealth helped them reach more rural Veterans with WH courses, which are paramount for learning about WH and constructing personalized health plans, instead of requiring the Veteran to visit a VA facility:

*…since COVID-19 hit, [we] have been doing a lot of “Intro to Whole Health” and the other courses, all through VVC [the VHA secure video telehealth platform]. And there I’ve gotten more participation rather than people coming in for the two hours [to complete the course]. (FG #3)*

Additionally, for some coaches, coaching virtually seemed to increase accessibility for rural Veterans living an appreciable distance from a facility, allowing for greater participation. One participant described:

*Some of our Veterans live two and a half to four hours away, and to commit to just a two-hour gathering and/or a once-a-week-for-six-weeks commitment is a lot for them. So obviously, we were working on, and are still continuing to work on, providing that via VVC through either CBOCs [VHA rural community-based outpatient clinics] or allowing them to access from home. (FG #3)*

#### 3.1.2. Overcoming geographic barriers

WH coaches reported that geographic barriers were a continuous challenge for many rural Veterans who find it difficult to reach VA facilities due to travel distance and who may not have internet:

*…many times, our Veterans feel like the door has just been closed in their face because [they say] ‘I can’t drive, I can’t get there, I don’t have internet’. (Interview ID #3)*

Participants expressed that telehealth helped some Veterans overcome geographic barriers, thereby increasing access for rural-residing Veterans. One coach explained that virtual WH has provided more options for attending programs or accessing services that rural Veterans might not have been able to because of distance from clinics, transportation challenges, or inability to attend in-person:

*…what I love about it is those rural Veterans now, who may not have come into the yoga or tai chi, now may have access to those…that they may not have had before. (Interview ID #15)*

Others mentioned that not needing to travel for WH coaching was another advantage of telehealth. For example, one participant noted that rural Veterans appreciated:

*[the] ability to meet virtually for coaching services instead of traveling to a facility for care. (Survey ID #18)*

Telehealth was thus seen as a more feasible option to reach some Veterans who might otherwise not be seen due to geographic barriers.

#### 3.1.3. Convenience and flexibility

Telehealth coaching also allowed for increased flexibility in scheduling that is not always available with in-person visits. This permitted appointments to be arranged at times convenient for the Veteran and interspersed with appointments at the VHA:

*…I don’t have to necessarily wait for your nutrition appointment at 3…I can call you at 8:30 in the morning and still have a coaching conversation or coaching session with you. (Interview ID #13)*

Additionally, telehealth allowed for Veterans to participate in WH in the comfort of their own homes, adding to its convenience. One coach reiterated that some WH sessions with patients were facilitated by the ease of remote connection:

*…Whether you live 10 minutes away or an hour and a half away, it’s a little bit easier just to sit at home for a 30-minute briefing. (FG #3)*

Moreover, some coaches reported that telehealth was highly appreciated by Veterans during the onset of COVID-19; so much so that some expressed a desire to continue meeting remotely due to the convenience of a video call, even when pandemic restrictions loosened:

*We do almost everything Video Connect [VVC] now, to the point where a lot of our rural Veterans [are] like—Great, now that we’ve done it, when you all get back in [after COVID], in the clinics, we’re just going to keep doing this video. (FG #2)*

### 3.2. Telehealth Gaps for Rural Veterans

In addition to advantages, participants described several gaps in using telehealth with rural Veterans. These gaps included rural internet access and connectivity issues, differing levels of digital literacy, and receptivity to telehealth coaching.

#### 3.2.1. Rural internet access and connectivity issues

Several participants mentioned that the lack of reliable internet and appropriate bandwidth, limited access to Wi-Fi in Veterans’ homes, and frequent connectivity issues made telehealth with rural Veterans more difficult. As one survey participant noted:

*Veterans Video Connect (VVC) for rural vets has been a challenge, with some not having internet access. (Survey ID #79)*

Participants expressed the need to ensure sufficient bandwidth and steady Wi-Fi for WH coaching classes to reduce annoyances and facilitate more enjoyable experiences. Many issues related to internet, connectivity, and bandwidth gaps resulted from broadband services not reaching remote locations, making telehealth a less viable option for some. Because of this, some coaches felt they could not adequately meet all of the needs of their rural Veterans:

*…\ we’re not really meeting the needs of [all] our Veterans because they don’t have internet access. At the main facility, we offer yoga, tai chi…and different complimentary therapies. However, because of access and the distance, they don’t go that far [to the facility]. Without having the internet, they’re not able to participate in those as well. (FG #1)*

One coach expressed how technological issues and lack of bandwidth became an impediment to getting some of their most rural Veterans to participate in video telehealth:

*…the very rural Veterans, they’re lucky if they have cell phone coverage out there. Video coverage—forget it. They’re not going to have it. (Interview ID #1)*

#### 3.2.2. Differing levels of digital literacy

Participants explained that rural Veterans had differing levels of digital literacy which resulted in a gap in delivering WH coaching virtually. Some Veteran patients had difficulties logging into virtual appointments, using VVC, accessing emails with important information, and utilizing virtual resources available to them. One participant felt that limited digital literacy may have caused Veterans to be insecure about using telehealth technology:

*Some of them [Veterans] have been very hesitant in actually wanting to get on a video connect call. I think a lot of it was [because] they were technology challenged and they felt [a] little self-conscious about it…I think the biggest challenge was…being afraid to navigate the technology, or afraid to actually say that they don’t know how to navigate the technology. (Interview ID #15)*

A possible contributor to the levels of digital literacy and capabilities perceived by survey participants was the age of rural Veterans, who tend to be older and may find themselves on the other side of the digital divide in generational terms. Some expressed concern about how increased reliance on technology to deliver care created challenges for older Veterans:

*[There is] more reliance on web-based applications that conflicts with the abilities of [the] generally elderly rural Veteran population. (Survey ID #5)*

When discussing telehealth for coaching with older rural populations, participants noted that these Veterans experienced difficulties using the technology and figuring out how to navigate equipment necessary for telehealth sessions:

*…the majority of Veterans that I dealt with were either more frontier [rural]…or they were older and didn’t…know how to work their phone via VVC [Veteran Video Connect] or didn’t want to take the time to figure out how to work their phone for VVC…the bandwidth didn’t work right, or they couldn’t figure out their camera, or they couldn’t figure out their microphone, or they just flat out didn’t have a computer … and they just didn’t like that. (Interview ID #14)*

#### 3.2.3. Receptivity to video telehealth coaching

Coaches felt that rural Veterans had varying degrees of receptivity to using video telehealth as a coaching modality. In some cases, this was attributed to rural Veterans initially being less receptive to the idea of telehealth coaching. Several survey participants mentioned that some rural Veterans simply did not want to have virtual appointments or use telehealth, demonstrating less receptiveness to interact through technology:

*[Veterans expressed] unwillingness to use the technology. (Survey ID #32)*

Other coaches felt that telehealth coaching may not always be the most favorable modality for rural Veterans, comparing it to in-person coaching which might be more appealing to some:

*With the current pandemic, we offer more video options for Veterans. Outside of the pandemic, the video options were limited and not as desirable for the Vets. At the end of the day, nothing beats face-to-face interaction with Veterans, and rural Vets often are not interested [in telehealth]. (Survey ID #17)*

Other participants noted that receptivity depended on the Veteran. Although some are receptive, there are those who might never buy into remote delivery of care due to a need for the kind of connection that is perceived only to be garnered in person. One coach felt that there was a struggle for some Veterans to participate in remote coaching, whether by telephone or video call:

*Sometimes…we have technical difficulties…sometimes Veterans get a little bit like, ‘ugh, this doesn’t even work’…But I think that a lot of them are willing. Some are just never really gonna do it. I mean they’re like ‘no, not anything about the computer, I’m not touching it’. They wanna come in…they’re so used to seeing their providers…and they think they need that connection. (Interview ID #12)*

### 3.3. Strategies for Bridging Telehealth Gaps

Participants emphasized strategies that could help address experienced telehealth gaps centering on technological challenges of telehealth delivery. Subthemes included increasing Veterans’ technological literacy, providing VHA-issued devices and virtual platforms, and increasing access to technical support for Veterans and coaches.

#### 3.3.1. Increasing Veterans’ technological literacy

Several participants explained that teaching rural Veterans about using technology could help overcome telehealth gaps they experience regarding digital literacy and facilitate telehealth coaching engagement. Some suggested offering classes to increase rural Veterans’ knowledge about accessing virtual appointments and coaching sessions and using the VVC app:

*Training for Vets on use of technology resources. I use a lot of phone visits to compensate for [Veteran] lack of comfort/access with VVC [Veteran Video Connect]. (Survey ID #66)*

In addition, a basic level of computer skills was important for WH coaching. Given that some rural Veterans may have lower levels of digital literacy and less experience using technological equipment, several survey participants recommended developing rural Veterans’ computer skills to increase their capabilities and confidence with using technology:

*We need some way of teaching our elder Veteran population how to utilize technology. A lot of them are rural and have never used a computer. (Survey ID #4)*

A couple participants also suggested going directly to the Veterans’ homes to help develop their technology skillset:

*For those Vets who are not computer savvy, you could find a fellow Vet in the area to go over and be a mentor for a day on technology…that would have a great impact. (Survey ID #34)*

Other participants mentioned equipping Veterans with tips and tricks as a strategy to help with troubleshooting connectivity issues:

*Slowing down my speaking and asking them about how do you hook up [to the internet]? Are you by a window? Do you have a place in your home that would face a window or the south…I was telling them to look in your neighborhood… wherever the disks [satellite dish] are that people have their DirecTV facing—maybe that’s the window you should sit in when you’re trying to connect with me. So, trying to give them some technical tips to see if that works. (Interview ID #11)*

Coaches emphasized the importance of bolstering Veterans’ technological problem-solving skills by both increasing their speech intelligibility and providing them with potential solutions for increasing connectivity.

#### 3.3.2. Providing VHA-issued devices and virtual platforms

Providing VHA tablets with a preloaded VVC application on the home screen, and hotspots, cell service, and/or Wi-Fi capabilities was mentioned by many participants as aspects of VHA’s telehealth infrastructure that helped with coaching. These devices were especially necessary for Veterans who might have Wi-Fi but lack equipment to participate in telehealth coaching:

*Being able to offer tablets to Veterans who have Wi-Fi but no equipment to attend virtual classes. (Survey ID #50)*

One participant emphasized the value of getting tablets into the hands of rural Veterans because these devices alleviated the need to travel or coordinate transportation on the Veteran’s end while still allowing the Veteran to access coaching services:

*Then we look at the digital divide [consult] to get them a VA-issued iPad… [we try] to get an iPad into our patient’s hands to do video appointments…to avoid travel, to avoid those things that come up for transportation…(Interview ID #3)*

Although VHA devices were noted as important for overcoming telehealth gaps, participants expressed those devices should be more readily accessible to Veterans. Similarly, several participants suggested there needs to be a more streamlined process for accessing devices:

*[We need] a much simpler process to obtain access to tablets for those who do not have a smartphone or computer. (Survey ID #42)*

Additionally, a couple participants suggested it would be beneficial to provide Veterans with WH coaching resources by offering YouTube videos of classes or developing virtual platforms where WH content was accessible:

*Perhaps a single website with access to all VA Integrative & Complementary health resources (YouTube yoga channels, mindful moments audio files, exercise videos, gratitude prompts, cooking classes for diabetics, etc.). We can then share with our patient population (as well as our employees). (Survey ID #44)*

A salient aspect of providing devices and resources to rural Veterans reported by most participants was making sure the technology was reliable and could be consistently and easily used.

#### 3.3.3. Increasing access to technical support for Veterans and coaches

Participants highlighted the importance of increasing access to technical support for both Veterans and WH coaches. They emphasized the need for knowledgeable, designated teams to help with installing equipment and provide guidance for technology-related issues or when complications arise:

*Digital divide consults (iPads) WITH an install, not just delivery of an iPad via mail. If there were teams that did house calls, much like UNIPER [telehealth and social engagement company] installs. (Survey ID #17)*

One participant explained how the presence of a strong telehealth team alleviated many of the struggles they had with engaging Veterans virtually, noting the importance of having support available to help with VHA device setup:

*We still have some challenges, but we also have a whole telehealth team that we don’t even have to help the Veterans set up. Once they are identified as [needing] telehealth services, we send in a consult to telehealth, and they’re the ones that send out an iPad and walk them through the setup. (FG #1)*

Another coach felt unprepared to teach patients how to use telehealth technology and how to walk them through potential solutions. They explained that trainings to improve coaches’ readiness to troubleshoot telehealth problems would be beneficial:

*I feel like I could be trained more in the process of [telehealth troubleshooting]… it’s one thing to know how to do it yourself and it’s another thing to know how to teach it…‘cause I’m like ‘the three dots, click the three [dots]’ and they’re like ‘what three dots?’ So, I feel like if there was a trainer type of thing for telehealth, I would definitely think that was beneficial to be able to learn how to teach someone…I don’t know how to do the troubleshooting thing. (Interview ID #8)*

#### 3.3.4. Leveraging low-tech modalities

Many participants indicated that video telehealth calls are ideal for WH coaching. However, due to infrastructural deficits, video telehealth is not always effective or the right fit for rural Veterans. In addition to video telehealth, participants spoke of delivering WH coaching through complementary, low-tech modalities based on the Veterans’ needs and specific situation. Participating coaches stressed the importance of leveraging non-internet-reliant options as a less cumbersome adjunct to video telehealth.

##### 3.3.4.1. Telephone-based coaching

One of the frequently discussed complementary modalities was telephone coaching instead of face-to-face or video telehealth. Participants reported this lower tech option was preferred by some Veterans. Some coaches noticed Veterans were more willing to try WH coaching when they could do it *via* telephone:

*I noticed more positive feedback and more positive interest in Whole Health when I switched to doing telephone appointments versus face-to-face. Which I know that sounds odd, but we got a lot more willingness or openness to everything including all the education pieces about the Whole Health… more so when it’s one-on-one with me on the phone, versus when we were in a group setting doing the Intro to Whole Health. (FG #3)*

Another coach mentioned they rely mostly on phone calls for their WH sessions, whether it be because some Veterans do not prefer VVC or because they cannot connect *via* video:

*I’ve been doing most of my coaching through phone. I would say probably about 80% of it’s through phone. And some people…they’ll [try to] do video and then they won’t connect, and we’ll just cut right over to the phones. (Interview ID #13)*

Other participants explained that telephone sessions could be used to overcome connectivity issues that make using telehealth difficult, for those who lack reliable internet, compelling some Veterans to seek a phone session in place of video telehealth coaching:

*I will tell you, post-COVID, … there’s a push for us to do video appointments. But a lot of my rural Veterans are like—‘Yeah, just call me. I just want to do this over the phone. I’m not interested in trying to figure all of that out.’ I’ve had a couple who have said that they just don’t have great internet service, so they would rather a phone call. (FG #2)*

Participants also shared that telephone calls can be an easier modality for rural Veterans to access and navigate as they require less technological skills and less understanding of VVC. This allowed for rural Veterans who experienced technology-related challenges to still partake in WH coaching:

*Often rural veterans that I work with have more tech challenges, so we meet via phone rather than VVC. (Survey ID #12)*

Not only were rural Veterans interested in utilizing telephone calls for WH coaching, but some participants also expressed a preference for coaching over the phone. They explained it was more convenient and allowed for easier access to Veterans:

*…we had a meeting and I said ‘once COVID stops, are we still allowed to continue to add the phone calls?’ ‘cause to me it’s a lot easier just to pick up the phone and have a coaching conversation right there versus them checking in, try and find them in a hallway…with everybody there or they forget… or then I’m waiting in the hall trying to find them… as much as I love face to face, right? (Interview ID#13)*

##### 3.3.4.2. DVDs and mailouts

Other modalities to engage rural Veterans in WH coaching mentioned by survey respondents and a FG participant included DVDs and mailouts. These were suggested as resources that could help rural Veterans overcome barriers related to internet or connectivity issues:

*[Something] that would help would be…having resources for those that don’t have internet connection- Tai Chi and yoga DVDs to mail out. (Survey ID #30)*

One FG participant mentioned that some Veterans did not have DVD players, which was a barrier they believed could be addressed:

*We were offering tai chi here in my clinic as a pilot. I would have four to six Veterans in my conference room, being led by the instructor from [city]…after so many weeks, [the Veterans] would get a DVD for improving their home practice. But we have noticed that a lot of them don’t even have a DVD player. And those DVDs, I think would be very helpful…I’d be willing to see if I could help with working past the barriers of technology….and getting our Veterans a DVD player, just to be able to play. (FG #1)*

This was something they were willing to further pursue as a way to overcome technological barriers faced by rural Veterans, demonstrating their support for this lower tech modality.

Mailouts were viewed as an especially useful modality to introduce Veterans to WH, get them interested in coaching, and begin building relationships:

*[Getting] them even willing to be coached…Or even participate in a coaching session…it tends to be more steps to get there…not only am I mailing out…more materials. There might be some other things that I send out just to create that relationship. (Interview ID #8)*

Other coaches mentioned mailing WH welcome kits and information packets, as well as follow-up letters, to gauge the Veteran’s interest in continuing with WH coaching.

#### 3.3.5. Persisting relevance of in-person visits for rural Veterans

Lastly, participants reiterated that there is a need for both virtual and face-to-face programs at VHA going forward, with in-person persisting as a relevant coaching modality. Participants explained that in-person interactions with rural Veterans was an important aspect of the WH coaching process. It allowed them to provide information about what WH entails, have authentic conversations, and address Veterans’ questions or concerns. While telehealth helped to extend coaching options, some participants reported that for some Veterans, the motivation for engaging in WH offerings was the direct interactions it provided:

*There’s a few [Veterans], there’s people that need in person. They need to see you. They need that…I can’t wait to get back to in person [after COVID-19], and hearing the stories in person, like I said, ‘cause you see the body language, you see the facial expressions…when part of the story is hard for them. (Interview #8)*

Several participants also mentioned that engaging with rural Veterans in-person was a great way to initially reach them with WH coaching. It was also a strategy for facilitating familiarity, authenticity, and trust and opened the door to the possibility of future telehealth coaching sessions:

*In-person meetings—allows a human connection which paves the way for virtual meetings. (Survey ID #56)*

Further, while video telehealth has been rewarding in permitting more rural Veterans to be seen, still some Veterans continued to prefer in-person visits. One participant expressed that coaching could take place across different modalities and had a place within the coaching repertoire, but in-person coaching was still preferred by some Veterans:

*I have to say the switch to video has been just as rewarding…I feel that it’s even more rewarding because I’m seeing more patients from all over…while still having the face-to-face feel [via video]…/…[but] as a health coach,…you could definitely have in-clinic days, you could have out-of-office days working remotely, and there are some [Veterans] that you have to see face-to-face, that have pushed off coaching because they don’t wanna live in a virtual world…but there is a time and place for every modality in coaching. (Interview ID #3)*

## 4. Discussion

Following the onset of the COVID-19 pandemic, the proportion of WH coaching efforts *via* telehealth increased in an unprecedented way. Telehealth, in the era of the COVID-19 pandemic, became a critical WH practice for engaging rural Veterans in WH care. Findings from this evaluation reveal that video-enabled telehealth became more mainstream in coaches’ delivery of WH. Still, almost all coaches in this evaluation reported an array of challenges while utilizing telehealth to engage with rural Veterans. To overcome these challenges and bridge telehealth gaps, WH coaches have integrated multiple modalities into their coaching practice; for example, using in-person, video telehealth, and non-internet reliant options, such as telephone, DVDs, and mail outs. Moreover, rather than employing a “one-size-fits-all” approach, our findings suggest that coaches should apply a pragmatic and blended approach of various modalities based on the Veterans’ contexts, needs, and preferences, which exemplifies a patient-driven approach to care (16, 19, 37, 38).

Research has noted that using telehealth modalities to remotely deliver clinical and rehabilitation services to rural Veterans has many advantages over in-person delivery (11–13, 38, 39). We confirm and expand upon these findings by demonstrating that virtual coaching with rural Veterans helped to overcome geographic and access barriers due to lack of travel and reduced distance to care, and provided flexibility to both coaches and patients (38, 40). Additionally, an increased online capacity made it easier, and more efficient, for coaches to deliver group sessions to more Veterans at one time and to provide opportunities to attend different types of integrative therapies from home (i.e., yoga, Tai Chi, other integrative therapies). These advantages align with the ethical principles of autonomy, distributive justice, and beneficence for the ethical practice of telehealth (38, 41).

However, all coaches in this study reported several existing gaps within the telehealth infrastructure at VHA and in the rural community. For example, the lack of reliable internet access and broadband internet in rural areas has caused frustration among coaches and hesitancy to participate *via* telehealth among rural Veterans. Challenges among some Veterans, especially older Veterans (1, 9), to adeptly use telehealth technology and devices such as an iPad or a computer adds to the frustration of both parties. Participants reported that these frustrations have led to varying degrees of receptivity toward telehealth services by some of their rural Veterans. Several other studies on the rapid transition of healthcare delivery during the pandemic era also reported similar patient- and system-level challenges that have impacted the success of service delivery *via* telehealth (38, 42, 43).

As digital forms of telehealth continue to be rolled out, ensuring equitable access to technology is not only a necessity but also ethically important. Establishing reliable broadband infrastructure for rural and other underserved populations must be addressed so as to not exacerbate the already existing digital divide (44) and health disparities among rural vs. urban and older vs. younger Veterans (4, 37, 38, 45). There are many opportunities to bridge these gaps. For example, the VHA should continue to focus on ensuring that a variety of modalities for healthcare delivery are available to improve access to coaching. Additionally, the VHA should continue to expand and streamline efforts to equip eligible rural Veterans with VHA-issued devices that have Wi-Fi capabilities (4, 14, 46).

As supported by Keenan et al., some of the experienced telehealth gaps were attributed to the rapidity of the transition to telehealth in response to the COVID-19 pandemic (38). Continued implementation of designated telehealth teams with the purpose of providing training and support to both coaches and Veterans would improve the Veteran’s access to, and satisfaction with, the remote care (38, 45). With strong infrastructural support within VHA for these recommended changes, WH coaching can continue to be an initiative well suited for telehealth delivery. Future studies are warranted to examine the impact of increased telehealth delivery and how to approach equitable access to telehealth in a way that does not favor one group over another (38, 41, 45).

Amid higher tech advancements and implementation, it is important to also acknowledge the advantages of lower tech modalities for WH coaching (37). Telephone health coaching was highlighted as a preferred approach for WH delivery by many participants due to its convenience, suitability, and easy availability among rural Veterans. This aligns with health coaching research in other fields that has found telephone coaching is valued by patients and coaches and overcomes the need to travel for those residing in rural areas (39, 47, 48). Telephone coaching has also been shown to be cost-effective due to low costs associated with its delivery (39, 47). However, phone appointments can sometimes receive less workload credit than video telehealth or in-person care. Therefore, it is critical to advocate that telephone coaching remains a billable form of contact so that more coaches are willing to utilize this option with those Veterans who prefer this modality over others (37) or who may have to rely on phone-based telehealth services due to undependable or inconsistent video connectivity.

Further, absence of optimal telehealth infrastructure as reported by many of our coaching participants makes it critical to preserve the value of in-person and telephone engagement (38). Findings from our study support a blended model of WH coaching where the initial contact and enrollment is done *via* face-to-face engagement followed by telephone or video telehealth coaching, working with the patient to develop the best blend of modalities to achieve their health goals. This is in line with other reports that have found health coaching to result in favorable outcomes when using a combination of modalities (37–39, 43).

To enhance WH use, there is a need for more infrastructural support for coaches to incorporate multiple approaches into their WH delivery. The VHA has pioneered initiatives to address the digital divide experienced in rural areas (44) and develop infrastructure for optimum delivery of health services among rural Veterans (14). As this continues, it will be important for researchers to examine which initiatives are most successful and sustainable for meeting the needs of rural Veterans, considering the ethical implications of telehealth practice. Moreover, while telehealth continues to be regarded as a key solution for meeting the needs of hard-to-reach, geographically dispersed areas, future studies are needed to critically assess the ethical and pragmatic implications telehealth delivery might have on the Veterans’ experience of WH (49).

## 5. Limitations

This project was designed to understand the WH coaches’ perspectives only and therefore non-coaches’ perspectives were not included. To ensure continuity of care for telehealth, it is important to adopt feedback from other groups regarding quality of telehealth utilization so that we can continue developing a strong telehealth infrastructure in VHA. Future studies could incorporate Veterans’ perspectives and non-coaches, such as clinicians who are increasingly incorporating aspects of WH coaching into their work at the VHA. Given the complexity of the WH program and the variety of stakeholders that are involved, a broader examination of this topic with all stakeholders is required to get a complete picture of the challenges and barriers to virtual WH coaching.

## 6. Conclusion

Amidst the expansion of telehealth options, our findings highlight that video telehealth alone is not sufficient for meeting the needs of rural Veterans. We recommend a blended approach of complementary modalities for WH coaching engagement and delivery, utilizing in-person, virtual, and lower tech options. However, we emphasize the fact that despite current challenges, telehealth is indeed now a viable option to provide WH coaching and other health care services to rural Veteran population and shows promise for reducing rural Veterans’ health access gaps and improving continuity of care. Of importance is engaging in shared decision-making about what type of modality fits each Veteran based on their living context and individual capabilities. We would also emphasize the importance of adopting an approach that is ethical and aimed to reduce and not augment the existing digital divide and health disparity among Veterans.

## Data availability statement

The data analyzed in this study is subject to the following licenses/restrictions: The datasets presented in this article are not readily available because supporting data cannot be made openly available due to privacy requirements. Requests to access these datasets should be directed to corresponding author.

## Ethics statement

This project was deemed a quality improvement project in accordance with VA guidance (ORD Program Guide 1200.21, “VA Operations Activities that May Constitute Research”) and in partnership with Office of Patient Centered Care & Cultural Transformation (OPCC&CT). According to VA quality improvement guidelines, ethical review, approval, and written informed consent were not required.

## Author contributions

JH-G and GC: project design and funding acquisition. JH-G, GC, CMK, KL, DV, CK, DCR, and HJ: conceptualization. JH-G, GC, and CMK: acquisition of data. JH-G, CMK, GC, KL, and DV: methodology. JH-G, DV, KL, and CK: analysis and interpretation of data. JH-G, KL, and CK: validation and drafting of initial work. JH-G, GC, and CK: resources and project administration. JH-G, DV, CMK, KL, GC, DCR, and HJ: critical appraisal and review, edits, and revisions. JH-G, DCR, and HJ: supervision. All authors contributed to the work and provided final approval of the version submitted for publication and agreed to be accountable for all aspects of the work as presented.

## Funding

The work was supported by the Office of Rural Health, U.S. Department of Veterans Affairs (Project ID: PROJ-03536).

## Conflict of interest

The authors declare that the research was conducted in the absence of any commercial or financial relationships that could be construed as a potential conflict of interest.

## Publisher’s note

All claims expressed in this article are solely those of the authors and do not necessarily represent those of their affiliated organizations, or those of the publisher, the editors and the reviewers. Any product that may be evaluated in this article, or claim that may be made by its manufacturer, is not guaranteed or endorsed by the publisher.

## Author disclaimer

The views expressed in this article are those of the authors, and no official endorsement by the U.S. Department of Veteran Affairs or the U.S. government is intended or should be inferred. The contents of this article do not represent the views of the Veterans Health Administration Office of Rural Health, the Department of Veterans Affairs, or the United States Government. Any opinions, findings, or conclusions expressed in this material are those solely of the authors.
